# Increasing genome instability in adrenocortical carcinoma progression with involvement of chromosomes 3, 9 and X at the adenoma stage

**DOI:** 10.1038/sj.bjc.6690748

**Published:** 1999-10

**Authors:** A J Russell, J Sibbald, H Haak, W N Keith, A M McNicol

**Affiliations:** University Department of Pathology, Royal Infirmary, Castle Street, Glasgow, G4 0SF, UK; Department of Internal Medicine, Diaconessenhuis, Eindhoven, The Netherlands; CRC Department of Medical Oncology, Glasgow, UK

**Keywords:** adrenal cortex, neoplasm, interphase cytogenetics, chromosomes 3, 9, X, imbalance

## Abstract

The investigation of chromosomal aberrations in adrenocortical tumours has been limited by the difficulties of applying classical cytogenetics to tumours with low levels of proliferation. We have therefore applied the technique of interphase cytogenetics to paraffin-embedded archival specimens of 14 adrenocortical adenomas and 13 carcinomas. Hybridizations were performed using centromere-specific probes to chromosomes 3, 4, 9, 17, 18 and X, which have been shown to be altered in other types of tumours. Chromosomal imbalance was defined on the basis of changes in both chromosome index (CI) and signal distribution (SD). Where only one of these was altered, this was classified as a tendency to gain or loss. On the basis of the analysis of optimal hybridizations, carcinomas showed gains in all chromosomes studied, five of nine showing gains in multiple chromosomes. Gains were most common in chromosomes 3, 9 and, in particular X, eight of 11 showing gain, and one a tendency to gain. Chromosomal gain was seen less commonly in adenomas, but again chromosomes 3, 9 and X were involved. Losses were infrequent, only one carcinoma showing loss of chromosome 18, and adenomas showing a tendency to loss of chromosomes 4 (two cases), 17 (one case) and 18 (two cases). Our data suggest that changes in chromosomes 3, 9 and X are early events in adrenocortical tumorigenesis, and that there is increasing chromosomal instability with tumour progression. © 1999 Cancer Research Campaign


*British Journal of Cancer* (1999) **81**(4), 684–689
© 1999 Cancer Research Campaign
Article no. bjoc.1999.0748

### Increasing genome instability in adrenocortical carcinoma progression with involvement of chromosomes 3, 9 and X at the adenoma stage

**AJ Russell[1], J Sibbald[1], H Haak[2], WN Keith[3] and AM McNicol[1]**

[1]University Department of Pathology, Royal Infirmary, Castle Street, Glasgow G4 0SF, UK; [2]Department of Internal Medicine, Diaconessenhuis, Eindhoven, The Netherlands; [3]CRC Department of Medical Oncology, Glasgow, UK

**Summary** The investigation of chromosomal aberrations in adrenocortical tumours has been limited by the difficulties of applying classical cytogenetics to tumours with low levels of proliferation. We have therefore applied the technique of interphase cytogenetics to paraffin-embedded archival specimens of 14 adrenocortical adenomas and 13 carcinomas. Hybridizations were performed using centromere-specific probes to chromosomes 3, 4, 9, 17, 18 and X, which have been shown to be altered in other types of tumours. Chromosomal imbalance was defined on the basis of changes in both chromosome index (CI) and signal distribution (SD). Where only one of these was altered, this was classified as a tendency to gain or loss. On the basis of the analysis of optimal hybridizations, carcinomas showed gains in all chromosomes studied, five of nine showing gains in multiple chromosomes. Gains were most common in chromosomes 3, 9 and, in particular X, eight of 11 showing gain, and one a tendency to gain. Chromosomal gain was seen less commonly in adenomas, but again chromosomes 3, 9 and X were involved. Losses were infrequent, only one carcinoma showing loss of chromosome 18, and adenomas showing a tendency to loss of chromosomes 4 (two cases), 17 (one case) and 18 (two cases). Our data suggest that changes in chromosomes 3, 9 and X are early events in adrenocortical tumorigenesis, and that there is increasing chromosomal instability with tumour progression. © 1999 Cancer Research Campaign

**Keywords:** adrenal cortex; neoplasm; interphase cytogenetics; chromosomes 3, 9, X; imbalance

Until recently, adrenocortical tumours were recognized clinically only if they secreted excess hormone or demonstrated obvious malignant behaviour. However, adrenocortical nodules of 2–3 cm diameter may be found at autopsy in 1.5–8.7% (Lack et al, 1990) of an unselected population. While some of these represent hyperplastic regenerative nodules adjacent to ischaemic atrophic change (Dobbie, 1969; Sasano et al, 1971), many are neoplastic. With the increasing use of sensitive scanning techniques, they are now being identified when patients are investigated for other intra-abdominal pathology. There is a therapeutic issue therefore of whether to remove them or not, because of the risk of malignancy (Prinz et al, 1982; Thompson and Cheung, 1987). Since carcinomas are on average larger than adenomas, at present larger lesions are removed, while smaller ones are monitored. However, in view of the extremely poor prognosis of adrenocortical carcinoma with mortality up to 92% (Karakousis et al, 1985) it would be important to develop markers of malignant potential which could be applied to individual lesions at the earliest possible stage, as even small lesions may be malignant (McNicol, 1992). This might be made possible by identifying specific molecular genetic events differentiating neoplastic from hyperplastic nodules and indicating malignant potential.

The molecular pathogenesis of adrenocortical tumours is poorly understood, and it is unclear whether there is an adenoma-carcinoma sequence. The rarity of the tumour and the low level of proliferation have made classical cytogenetic analysis difficult. Demonstration of allelic loss on chromosomes 11p, 13q and 17p (Henry et al, 1989; Yano et al, 1989) suggest that genes at these loci may have a role in pathogenesis. Familial adrenocortical cancer occurs in Beckwith–Wiedemann syndrome linked to 11p 15.5, and now known to be associated with paternal disomy for the insulin-growth factor-II (IGF-II) gene (Weksberg et al, 1993). It also occurs more frequently in association with the Li–Fraumeni syndrome (Li and Fraumeni, 1969), associated with germline mutations of the *p53* gene, on 17p13 (Srivastava et al, 1990). The demonstration of abnormal expression of IGF-II (Ilvesmaki et al, 1993) and p53 (Ohgaki et al, 1993; McNicol et al, 1997) in sporadic adrenocortical carcinoma would support a role for these proteins in tumorigenesis. The MEN-1 locus on chromosome 11q is another possible site of interest (Skogsaid et al, 1992; Iida et al, 1995). In an attempt to identify other potential loci, Kjellman et al (1996) have used comparative genomic hybridization (CGH) to screen for chromosomal gain and loss in adrenocortical tumours. Their data indicate that widespread changes can be found in carcinoma, but are rare in adenoma.

We have chosen the alternative approach of interphase cytogenetics (Poddighe et al, 1992), where probes specific to individual chromosomes are hybridized to paraffin sections. This has the advantage of allowing the analysis of changes in chromosome number in defined tumour cell populations (Murphy et al, 1995). The aims were to define and compare chromosome imbalances in a series of adrenocortical adenomas and carcinomas. This would potentially permit us to identify loci of interest in tumour pathogenesis and progression. The chromosomes selected for analysis have previously been shown to be altered in other tumours.

*Received 8 September 1998*
*Revised 20 November 1998*
*Accepted 26 January 1999*

*Correspondence to:* AM McNicol

A B

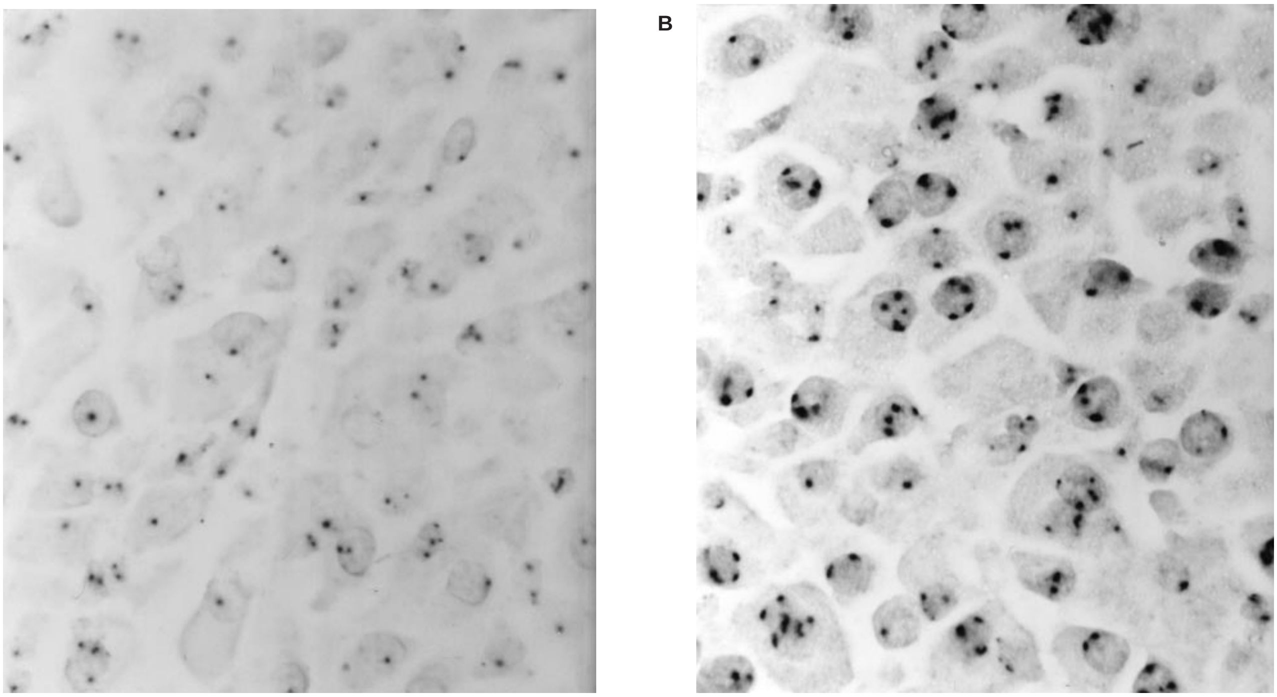

**Figure 1** (**A**) Adrenocortical carcinoma hybridized with centromeric probe for chromosome 18, showing two copies in most cells. (**B**) Adrenocortical carcinoma hybridized with a centromeric probe for chromosome 9 showing three or more signals in many cells

### MATERIALS AND METHODS

### Study population

Archival specimens were obtained from 14 adrenocortical adenomas and 13 carcinomas. These had been classified on the basis of clinical behaviour and on the histological criteria of van Slooten et al (1985). All tissues were fixed in formalin and embedded in paraffin wax. Normal human tonsil was used as control. A diffusely hyperplastic adrenal gland removed surgically from a patient with Cushing's disease was also included as a control.

### Tissue section preparation (Murphy et al, 1995)

In brief, sections, 6-μm thick, were mounted on glass slides coated in aminopropyltriethoxysilane. Before use, the slides were baked at 65°C for 4–24 h. The tissue sections were dewaxed, rehydrated and left to air dry. Sections were then digested with pepsin (0.4% pepsin in 0.2 N hydrochloric acid) for 5–60 min and post-fixed for 10 min in tissue fixative (Streck Laboratories Inc., Omaha, NE, USA). Sections were dehydrated for 2 × 3 min in 70% ethanol and 2 × 3 min in 100% ethanol, and left to air dry.

### DNA probes

Chromosome-specific repetitive sequence probes for the following loci were purchased from Oncor, Inc. (Gaithersburg, MD, USA): D3Z1 (chromosome 3), D4Z1 (chromosome 4), D9Z1 (chromosome 9), D17Z1 (chromosome 17), D18Z1 (chromosome 18), and DXZ1 (chromosome X). All commercial probes were ready labelled with digoxigenin. Probes were diluted in a hybridization mix consisting of 70% formamide, two times the standard concentration of standard saline citrate (SSC) (1 × SSC is 0.15 M sodium chloride and 0.015 M sodium citrate, pH 7), 500 μg $ml^{-1}$ salmon sperm DNA, and 10% dextran sulphate.

### In situ hybridization

The probe in the hybridization mix and DNA in a tissue section were denatured together using the Omnislide modular system (Hybaid Ltd, London, UK) at 80°C for 5 min, then incubated at 37°C overnight. After hybridization, slides were washed twice in 50% formamide and 1 × SSC at 42°C for 10 min and twice in 2 × SSC at 42°C for 10 min. Prior to immunocytochemical detection, slides were blocked for 30 min in 4 × SSC-TB (4 × SSC, 0.05% Tween-20, and 0.5% Boehringer blocking agent; Boehringer Mannheim, GmbH, Germany). Sites of hybridization were detected using anti-digoxigenin alkaline phosphatase (AP) Fab fragments (Boehringer Mannheim, GmbH, Germany) 1:300 dilution in 4 × SSC-TB, incubated for 45 min at room temperature. Slides were washed in 4 × SSC, 0.5% Tween-20 for 20 min, then rinsed in distilled water. The slides were then incubated in NBT/BCIP solution (75 mg $ml^{-1}$ nitroblue-tetrazolium (NBT), 50 mg $ml^{-1}$ 5-bromo-4-chloro-3-indolyl phosphate (BCIP)), 1.25 mM levamisole, 0.1 M Tris base, 0.1 M Tris–HCl, 0.1 mM sodium chloride, 50 mM magnesium chloride overnight. Sites of binding were identified as blue–black dots.

### Quantitation of hybridization signals

Chromosome-specific centromeric probes were hybridized to sections of adrenocortical adenoma and adrenocortical carcinoma

© 1999 Cancer Research Campaign as shown in Figure 1. In order to obtain control values, centromere copy numbers for the six chromosomes were assessed using tonsil tissue. Hyperplastic adrenal gave a similar pattern of hybridization. The evaluation and interpretation of ISH signals were carried out as described by Hopman et al (1992) and Murphy et al (1995) in which overlapping nuclei were not analysed, minor hybridization signals identifiable by their low intensity and smaller spot area compared with optimal control hybridizations were not counted. Poor quality hybridizations were not analysed. Only nuclei with the histological appearance of tumour cells were evaluated. For each section, the number of signal spots per nucleus was recorded for 200 nuclei. The hybridization data were analysed in two ways to assess the degree of chromosome imbalance for each sample and each chromosome. Firstly, the chromosome index (CI) was calculated (Dhingra et al, 1994) by dividing the total number of hybridization spots counted by the total number of nuclei counted. The CI gives an average chromosome copy number and is therefore better suited to describe clonal changes within a tumour. The CIs were calculated and plotted in Figure 2. The mean CI for normal tissue was 1.42, and three standard deviations from the mean gave values of 1.19 and 1.65. A tumour was defined as polysomic for a given chromosome if its CI was greater than 1.65. A tumour was defined as monosomic for a given chromosome if its CI was less than 1.19. Changes for X chromosome were analysed only in female cases.

The second method used to define chromosomal polysomy or monosomy is the signal distribution. A tumour was described as polysomic for a chromosome if the percentage of nuclei with more than two hybridization sites was greater than 10% of the nuclei counted. A tumour was described as monosomic for a chromosome if the percentage of nuclei with fewer than two hybridization sites was greater than 60% of the nuclei counted. The analysis of signal distribution can potentially detect relatively small populations of cells with numerical imbalances in chromosomes. These criteria for both signal distribution and CI were based on published estimates and previous experience of the technique and take into account nuclear truncation (Hopman et al, 1992; Macoska et al, 1993; Baretton et al, 1994; Dinghra et al, 1994; Murphy et al, 1995).

**Table 1** Tumours showing evidence of chromosomal changes by signal distribution

| | Chromosome number | | | | | |
|---|---|---|---|---|---|---|
| | **3** | **4** | **9** | **17** | **18** | **X** |
| Carcinomas | | | | | | |
| Gain | 4/9 | 4/9 | 5/10 | 3/9 | 2/9 | 5/6 |
| Loss | | | | | 1/9 | |
| Adenomas | | | | | | |
| Gain | 2/6 | 1/10 | 3/14 | 1/12 | | 2/10 |
| Loss | | 2/10 | | 1/12 | 2/9 | |

**Table 2** Chromosomal imbalances in adrenocortical carcinoma

| Chromosome no. | Loss | Balanced | Gain | Total |
|---|---|---|---|---|
| 3 | 0 | 6 | 3 (2) | 9 |
| 4 | 0 | 7 | 2 (2) | 9 |
| 9 | 0 | 5 | 5 | 10 |
| 17 | 0 | 6 | 3 | 9 |
| 18 | 1 | 6 | 2 | 9 |
| X | 0 | 3 | 8 (1) | 11 |

Chromosomal imbalance was assessed on the combination of chromosome index and signal distribution. Figures in brackets represent tumours showing imbalance by either measurement alone.

### Statistical analysis

Differences in chromosomal indices between adrenocortical adenomas and normal and carcinomas and normal were compared using the two-sided Mann–Whitney test with *a priori* level of statistical significance set at $P < 0.05$.

### RESULTS

In situ hybridization with chromosome-specific probes was carried out on 14 adrenocortical adenomas and 13 carcinomas, although

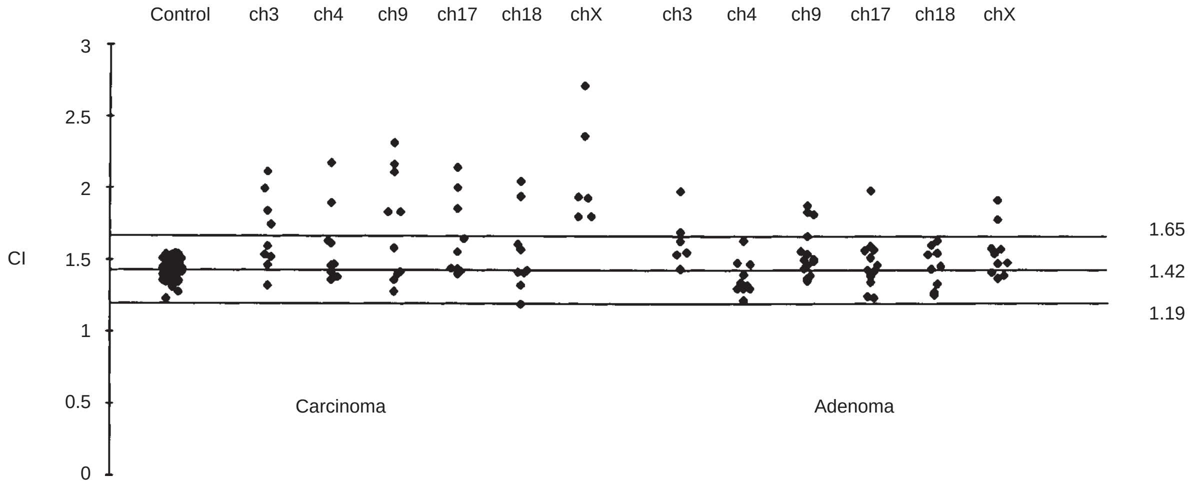

**Figure 2** Scattergram showing distribution of chromosome index (CI) for chromosomes 3, 4, 9, 17, 18 and X in a series of adrenocortical carcinomas and adenomas. The bars show the mean ± 3 s.d. of the controls. Values had to fall outwith these limits to be classed as gain or loss (ch = chromosome)

*© 1999 Cancer Research Campaign*

**Table 3** Chromosomal imbalances in adrenocortical adenoma

| Chromosome no. | Loss | Balanced | Gain | Total |
|---|---|---|---|---|
| 3 | 0 | 4 | 2 | 6 |
| 4 | 0 (2) | 10 | 0 (1) | 10 |
| 9 | 0 | 11 | 3 (1) | 14 |
| 17 | 0 (1) | 11 | 1 | 12 |
| 18 | 0 (2) | 9 | 0 | 9 |
| X | 0 | 9 | 2 | 11 |

Chromosomal imbalance was assessed on the combination of chromosome index and signal distribution. Figures in brackets represent tumours showing imbalance by either measurement alone.

**Table 4** Accumulation of chromosomal imbalances

| | No. of chromosome imbalances | | | | | | | |
|---|---|---|---|---|---|---|---|---|
| | 0 | 1 | 2 | 3 | 4 | 5 | 6 | Total |
| No. of adenomas | 5 | 3 | 0 | 0 | 1 | 0 | 0 | 9 |
| No. of carcinomas | 1 | 3 | 1 | 1 | 1 | 1 | 1 | 9 |

hybridization with all probes was not achieved on every tumour. Figure 1A shows an example of a section of adrenocortical carcinoma hybridized with a chomosome 18 probe showing two copies per cell, while Figure 1B shows hybridization with a chomosome 9 probe showing three or more signals in many cells.

### Chromosome index

The data are shown in Figure 2. Subjectively, there were frequent gains in all chromosomes tested in adrenocortical carcinomas, particularly for chromosomes 3, 9 and X. Losses were rarely identified, only one showing loss of chromosome 18. The visual changes were confirmed as significantly different from normal for chromosome 3 ($P$ = 0.002), chromosome 9 ($P$ = 0.048), chromosome 17 ($P$ = 0.025) and chromosome X ($P$ < 0.001). Subjective gains of chromosomes were seen in adenomas although less frequently than in carcinomas. These were confirmed as different from controls by statistical analysis in chromosome 3 ($P$ = 0.003) and chromosome X ($P$ = 0.027).

### Signal distribution

The data are shown in Table 1. Again, there appeared to be widespread gains in adrenocortical carcinomas, chromosomes X, 9, 3 and 4 showing most marked changes with evidence of one loss of chromosome 18. Adenomas showed fewer changes, with gains in chromosomes 3, 9 and X more marked. Losses were more commonly identified, again in 18, and also 4 and 17.

### Assessment of chromosomal imbalance

The data in Figure 2 were combined with analysis of signal distribution (Table 1) to assess chromosomal imbalance. The overall data are shown in Tables 2 and 3. The figures in brackets show the numbers of tumours with a trend to gain or loss where this was seen in only one measurement. In general, the pattern follows that seen in Figure 2. In carcinomas, gains were seen in all chromosomes tested. This was particularly prominent with chromosome X, eight of 11 showing gain while a further carcinoma showed a trend towards gain. No losses were identified, although one carcinoma showed a trend towards loss of chromosome 18.

The most common aberration in adrenocortical adenomas was gain of chromosome 9, two of 14 showing gain and a further adenoma a trend towards gain. Gains of 3, 17 and X were also seen. No losses were identified within the range of chromosomes studied, although adenomas showed a trend towards loss of chromosomes 4 ($n$ = 2), 17 ($n$ = 1) and 18 ($n$ = 2).

### Accumulation of chromosomal imbalances

Using the hybridization data for the six centromeric probes, each tumour was analysed for the total number of chromosome imbalances accumulated. Only those tumours which had been successfully tested for at least four of the six chromosome-specific centromeric probes were taken into account. Tumours had to show imbalance by both CI and signal distribution to be counted. Table 4 shows greater accumulation of chromosomal imbalances in adrenocortical carcinomas when compared to adrenocortical adenomas.

### DISCUSSION

This is the first study in which interphase cytogenetics has been applied to define changes in chromosomal copy number in a series of adrenocortical tumours. The technique is particularly useful because of the low level of proliferation even in carcinomas, rendering classical cytogenetic analysis difficult. A recent study has also suggested that the cells which grow in culture may not be representative of the total tumour population (Rosenberg et al, 1995). The chromosomes selected for study have been shown to have imbalances in other tumours. Our analysis was stringent, in that we defined chromosomal imbalance only if both CI and signal distribution agreed. In addition, our cut-off points were strict, with 3 standard deviations (s.d.) on CI, compared to 2.58 s.d. used in some other studies (Bulten et al, 1998) and a 60% level for cells showing fewer than two hybridization sites to define loss on signal distribution compared with 40% (Visscher et al, 1996) or 15% (Sneige et al, 1996). We may thus have underestimated the extent of gain or loss. Nevertheless, we demonstrated gains in all carcinomas, and in all chromosomes studied, although the pattern varied somewhat with each tumour. These changes did not simply reflect general changes in DNA ploidy, as the chromosome index varied with the individual chromosomes. The gains in 9 (50%) and X (72%) were particularly striking. Chromosomes 3, 4 and 17 also showed a significant level of gain, particularly when tumours showing a trend were considered. Where trends were recognized, this was usually due to a gain detected by signal distribution and not by CI. As discussed in Methods, this probably reflects the presence of subclones within the tumour. Endoreduplication followed by chromosomal loss and acquisition of structural chromosomal abnormalities is thought to be a feature of tumour development (Shackney et al, 1989; Cornelisse et al, 1992; Devilee et al, 1994). This may result in the emergence of subclones within a tumour which then progress through the accumulation of mutant genes conferring growth advantage (Shackney et al, 1989). These changes may reflect inactivation of tumour suppressor genes due to point mutations and loss of heterozygosity (LOH). Potential candidates include p53 on 17p (McBride et al, 1986). We (McNicol et al, 1997) and others

*© 1999 Cancer Research Campaign*

(Ohgaki et al, 1993) have shown altered p53 expression, most probably as a late event, in the development of adrenocortical carcinoma. There are two candidate loci on chromosome 18; DPC4 (deleted in pancreatic carcinoma) (Hahn et al, 1996) and DCC (deleted in colon cancer) (Vogelstein et al, 1988). Interestingly, the DPC4 gene encodes SMAD4, an important intracellular component of the signalling pathway for the inhibin/activin growth factor family (Kingsley, 1994). Recent evidence from inhibin-α knockout mice (Matzuk et al, 1994) and transgenic mice bearing an inhibin-α promoter Simian virus 40-T antigen transgene (Kananen et al, 1996) implicates the inhibin/activin family in adrenocortical tumorigenesis, with a suggestion that inhibin-α may be a tumour suppressor (Matzuk et al, 1994). Further investigation using microsatellite markers could be used to identify possible changes at these loci. There are other genes of potential interest on the chromosomes showing gains. Telomerase RNA which forms an integral part of the telomerase protein–RNA complex involved in immortalization is encoded by a gene on chromosome 3 (Feng et al, 1995). Interestingly, a recent report on a small number of cases suggests that the identification of telomerase activity in adrenocortical tumours may indicate malignant potential (Hirano et al, 1998). On chromosome 9, p16 is a candidate (Kamb et al, 1994). On chromosome X there is the DAX-1 gene, which is important in the differentiation and regulation of steroidogenic tissues (Guo et al, 1996).

Direct comparisons are not possible between the results of interphase cytogenetic studies and CGH (Ried, 1998). Using the alphoid repeat centromeric probes fine mapping of changes cannot be achieved. However, the CGH study on adrenocortical tumours also indicated widespread aberration in carcinoma (Kjellman et al, 1996), with gains and losses of all chromosomes, including those which we examined. Their study indicated that the extent of change increased with tumour size. We were unable to perform such an analysis because of lack of information on tumour size in our cases.

Our study suggests that chromosomal imbalance is much less common in adenoma than in carcinoma and that changes in chromosomes 3, 9 and X may occur at an early stage. The identification of changes only on signal distribution would suggest that clonal evolution is taking place. Common patterns seen in adenomas and carcinomas and the accumulation of chromosomal imbalances with tumour progression support the existence of an adenoma–carcinoma sequence. One adenoma showed multiple gains: this was a 13-year-old girl with a virilizing tumour who was disease-free after 12 years of follow-up. Whether this reflects the efficacy of surgery in this case or the lack of a critical step in tumour progression is unknown.

The widespread abnormalities defined in our study are therefore in keeping with the results of CGH which indicate that genetic aberrations are common in adrenocortical cancer. This would inevitably lead to major alterations in regulatory pathways and may help explain the aggressive nature and resistance to therapy of this tumour.

### REFERENCES

Baretton GB, Valina C, Vogt T, Schneiderbanger K, Diebold J and Lohrs U (1994) Interphase cytogenetic analysis of prostatic carcinomas by use of non-isotopic in situ hybridization. *Cancer Res* **54**: 4472–4480

Bulten J, Poddighe PJ, Robben JCM, Gemmink JH, de Wilde PCM and Hanselaar AGJM (1998) Interphase cytogenetic analysis of cervical intraepithelial neoplasia. *Am J Pathol* **152**: 495–503

Cornelisse CJ, Kuipers-Dijkshoorn N, van Vliet M, Hermans J and Devilee P (1992) Franctional allelic imbalance in human breast cancer increases with tetraploidization and chromosome loss. *Int J Cancer* **50**: 544–548

Devilee P, Schuuring E, van der Vijer MJ and Cornelisse CJ (1994) Recent developments in the molecular genetic understanding of breast cancer. *Crit Rev Oncol* **5**: 247–270

Dhingra K, Sneige N, Pandita TK, Johnston DA, Lee JS, Emami K, Hortobagyi GN and Hitletman WN (1994) Quantitative analysis of chromosome in situ hybridization signal in paraffin-embedded tissue sections. *Cytometry* **16**: 100–112

Dobbie JW (1969) Adrenocortical nodular hyperplasia. The ageing adrenal gland. *J Pathol* **99**: 1–18

Feng J, Funk WD, Wang S-S, Weinrich SL, Avilion AA, Chiu C-P, Adams RR, Chang E, Allsopp RC, Yu J, Le S, West MD, Harley CB, Andrews WH, Greider CW and Villeponteau B (1995) The RNA component of human telomerase. *Science* **269**: 1236–1241

Guo W, Burris TP, Zhang YH, Huang BL, Mason J, Copeland KC, Kupfer SR, Pagon RA and McCabe ER (1996) Genomic sequence of the DAX 1 gene: an orphan nuclear receptor responsible for X-linked adrenal hypoplasia congenita and hypogonadotropic hypogonadism. *J Clin Endocrinol Metab* **81**: 2481–2486

Hahn SA, Schutte M, Hoque TMS, Moskaluk CA, da Costa LT, Rozenblum E, Weinstein CL, Fischer A, Yeo CJ, Hruban RH and Kern SE (1996) DPC4, a candidate tumor suppressor gene at human chromosome 18q21.1. *Science* **271**: 350–354

Henry I, Grandjouan S, Couillin P, Barichard F, Huerre-Jeanpierre C, Glaser T, Philip T, Lenoir G, Chaussain J and Junien C (1989) Tumor-specific loss of 11p15.5 alleles in del 11p13 Wilms tumor and in familial adrenocortical carcinoma. *Proc Natl Acad Sci USA* **86**: 3247–3251

Hirano Y, Fujita K, Suzuki K, Ushiyama T, Ohtawara Y and Tsuda F (1998) Telomerase activity as an indicator of potentially malignant adrenal tumors. *Cancer* **83**: 772–776

Hopman AH, Poddighe P, Moesker O and Ramaekers FC (1992) In: *Diagnostic molecular pathology: a practical approach.* Vol 1. Herrington CS, McGee JO'D, eds. IRL Press, Arlington (VA). 141–167

Iida A, Blake K, Tunny T, Klemm S, Stowasser M, Hayward N, Gordon R, Nakamura Y and Imai T (1995) Allelic losses on chromosome band 11q13 in aldosterone-producing adrenal tumors. *Genes Chromosomes Cancer* **12**: 73–75

Ilvesmaki V, Kahri AI, Miettinen PJ and Voutilainen R (1993) Insulin-like growth factors (IGFs) and their receptors in adrenal tumors: high IGF-II expression in functional adrenocortical carcinomas. *J Clin Endocrinol Metab* **77**: 852–858

Kamb A, Gruis NA, Weaver-Feldhaus J, Liu Q, Harshman K, Tavtigian SV, Stockert E, Day RS, Johnson BE and Skolnick MH (1994) A cell cycle regulator potentially involved in genesis of many tumor types. *Science* **264**: 436–440

Kananen K, Markkula M, Mikola M, Rainio EM, McNeilly A and Huhtaniemi I (1996) Gonadectomy permits adrenocortical tumorigenesis in mice transgenic for the mouse inhibin alpha-subunit promoter/Simian virus-40-T-antigen fusion gene – evidence for negative autoregulation of the inhibin alpha subunit gene. *Mol Endocrinol* **10**: 1667–1677

Karakousis CP, Rao V and Moore R (1985) Adrenal adenocarcinomas – histologic grading and survival. *J Surg Oncol* **29**: 105–111

Kingsley DM (1994) The TGF-β superfamily: new members, new receptors, and new genetic tests of function in individual organisms. *Genes Dev* **8**: 133–146

Kjellman M, Kallioniemi O-P, Karhu R, Höög A, Farnebo L-O, Auer G, Larsson C and Bäckdahl M (1996) Genetic aberrations in adrenocortical tumors detected using comparative genomic hybridization correlate with tumor size and malignancy. *Cancer Res* **56**: 4219–4223

Lack EE, Travis WD and Oertel JE (1990) Adrenal cortical nodules, hyperplasia and hyperfunction. In: *Pathology of the Adrenal Gland,* Lack EE (ed), pp. 75–113. Churchill Livingstone: New York

Li FP and Fraumeni JF Jr (1969) Soft-tissue sarcomas, breast cancer and other neoplasms: a familial syndrome? *Ann Intern Med* **71**: 747–752

Macoska JA, Micale MA, Sakr WA, Benson PD and Wolman SR (1993) Extensive genetic alterations in prostate cancer revealed by dual PCR and FISH analysis. *Genes Chromosomes Cancer* **8**: 88–97

McBride OW, Merry D and Givol D (1986) The gene for human p53 cellular tumor antigen is located on chromosome 17 short arm (17p13). *Proc Natl Acad Sci USA* **83**: 130–134

 © 1999 Cancer Research Campaign

McNicol AM (1992) The human adrenal gland. Aspects of structure, function and pathology. In: *The Adrenal Gland*, 2nd edn, James VHT (ed), pp. 1–42. Raven Press Ltd: New York

McNicol AM, Nolan CE, Struthers AJ, Farquharson MA, Hermans J and Haak HR (1997) Expression of p53 in adrenocortical tumours: clinicopathological correlations. *J Pathol* **181**: 146–152

Matzuk MM, Finegold MJ, Mather JP, Krummen L and Bradley A (1994) Development of cancer cachexia-like syndrome and adrenal tumors in inhibin deficient mice. *Proc Natl Acad Sci USA* **91**: 8817–8821

Murphy DS, Hoare SF, Going JJ, Mallon EA, George WD, Kaye SB, Brown R, Black DM and Keith WN (1995) Characterization of extensive genetic alterations in ductal carcinoma by in situ by fluorescence in situ hybridization and molecular analysis. *J Natl Cancer Inst* **87**: 1694–1704

Ohgaki, H, Kleihues P and Heitz PU (1993) P53 mutations in sporadic adrenocortical tumors. *Int J Cancer* **54**: 408–410

Poddighe PJ, Ramaekers FC and Hopman AH (1992) Interphase cytogenetics of tumors. *J Pathol* **166**: 215–224

Prinz RA, Brooks MH, Churchill R, Graner JL, Lawrence AM, Paloyan E and Sparagana M (1982) Incidental adrenal masses detected by computed tomographic scanning – is operation required? *JAMA* **248**: 701–704

Ried T (1998) Interphase cytogenetics and its role in molecular diagnostics of solid tumors. *Am J Pathol* **152**: 325–327

Rosenberg C, Dellarosa VA, Latronico AC, Mendonca BE and Vianna Morgante AM (1995) Selection of adrenal tumor cells in culture demonstrated by interphase cytogenetics. *Cancer Genet Cytogenet* **79**: 36–40

Sasano N, Takezawa Y, Sato H and Horikawa N (1971) Microangiography of normal and pathological human adrenals in prenatal and aging course. *Tohoku J Exp Med* **104**: 129–141

Shackney SE, Smith CA, Miller BW, Burholt DR, Murtha K, Giles HR, Ketterer DM and Pollice AA (1989) Model for the genetic evolution of human solid tumors. *Cancer Res* **49**: 3344–3354

Skogseid B, Larsson C, Lindgren P, Kvanta E, Rastad J, Theodorsson E, Wide L, Wilander E and Öberg K (1992) Clinical and genetic features of adrenocortical lesions in multiple endocrine neoplasia type 1. *J Clin Endocrinol Metab* **75**: 76–81

Sneige N, Sahin A, Dinh M and El-Naggar A (1996) Interphase cytogenetics in mammographically detected breast lesions. *Hum Pathol* **27**: 330–335

Srivastava S, Zou Z, Pirollo K, Blattner W and Chang EH (1990) Germline transmission of a mutated p53 gene in a cancer-prone family with Li-Fraumeni syndrome. *Nature* **348**: 747–749

Thompson NW and Cheung PSY (1987) Diagnosis and treatment of functioning and nonfunctioning adrenocortical neoplasms including incidentalomas. *Surg Clin North Am* **67**: 423–436

Van Slooten H, Schaberg A, Smeenk D and Moolenaar AJ (1985) Morphologic characteristics of benign and malignant adrenocortical tumors. *Cancer* **55**: 766–773

Visscher DW, Wallis TL and Crissman JD (1996) Evaluation of chromosome aneuploidy in tissue sections of preinvasive breast carcinomas using interphase cytogenetics. *Cancer* **77**: 315–320

Vogelstein B, Fearon ER, Hamilton SR, Kern SE, Preisinger AC, Leppert M, Nakamura Y, White R, Smits AMM and Bos JL (1988) Genetic alterations during colorectal tumor development. *N Engl J Med* **319**: 525–532

Weksberg R, Teshima I, Williams BRG, Greenberg CR, Pueschel SM, Chernos JE, Fowlow SB, Hoyme E, Anderson IJ, Whiteman DAH, Fisher N and Squire J (1993) Molecular characterization of cytogenetic alterations associated with the Beckwith-Wiedemann syndrome (BWS) refines the localization and suggests the gene for BWS is imprinted. *Hum Mol Genet* **2**: 549–556

Yano T, Linehan M, Anglard P, Lerman M, Daniel L, Stein C, Robertson C, LaRocca R and Zbar B (1989) Genetic changes in human adrenocortical carcinomas. *J Natl Cancer Inst* **81**: 518–523

© 1999 Cancer Research Campaign
